# The occurrence of cancer in vertebrates: a mini review

**DOI:** 10.1186/s40709-020-00119-0

**Published:** 2020-06-08

**Authors:** Christos V. Kitsoulis, Athanasios D. Baxevanis, Theodore J. Abatzopoulos

**Affiliations:** Department of Genetics, Development & Molecular Biology, School of Biology, Faculty of Sciences, Aristotle University of Thessaloniki, 54124 Thessaloniki, Greece

**Keywords:** Neoplasia, Cancer, Animal taxa, Vertebrates, Evolution, Phenotype, Speciation

## Abstract

Neoplasia is a multilevel condition caused by irregularities over the genome, which can lead to a fatal result. To fully understand this phenomenon, an evolutionary challenge has risen during the last decades, away from human limits, driving the scientific quest into the wild life. The study of wild vertebrate populations in their natural habitats has shown that cancer is rather prominent. Thus, the diversity of vertebrates reported with some form of neoplasia is quite scattered through a variety of habitats. However, some species constitute exceptions by exhibiting cancer-protective features, driven by certain loci in their DNA. It is obvious that from an evolutionary standpoint, the incidence of cancer in different taxa is nowadays studied by seeking for patterns and their roots. The main purpose of the evolutionary approach is no other than to answer a fundamental question: Could cancer be ultimately regarded as another evolutionary force conducive to the formation or shaping-up of species?

## Introduction

Cancer is a multidimensional phenomenon in which a single cell or a group of cells are being involved in abnormal/non-adjustable growth. The resulting mass lesions are capable of following two different paths: they can remain benign or invade other parts of the body, as metastatic tumors. Cancer is a consequence of the organism’s multicellularity and a prime example of multilevel selection [1], i.e. cancer is a complex phenomenon driven by different types of selective forces. In order to gain a more holistic view of the disease, it is necessary to approach it by different angles and disciplines. Τo understand the disease, most research has focused on molecular and cellular processes in specific types of neoplasia [2]. So far, major lines of investigation, among others, include the pathology of the disease, its developmental stages, functional mechanisms of cancer cells and the polyclonality of tumors in an effort to formulate effective cure treatments. Over the last two decades, the prospect of studying cancer by exploring the relationship of the evolutionary process and the environment has emerged [ [3] and references therein]. Although the idea of considering cancer as an evolutionary phenomenon is not entirely new, little attention has been paid to the applications and assumptions of evolutionary biology for understanding neoplastic development [3]. More or less, the origin and progression of cancer are highly connected with the evolutionary concepts and the environment itself [1]. As a consequence, a growing number of scientists try to clarify what is going on about the appearance of cancer in higher taxa. However, understanding the complexity of the disease as a whole, dictates a multidisciplinary approach that may give new insights and prospects. As a result, it seems that much more attention has to be put on the wild populations and their natural habitat, of which very few items are known so far.

The evolutionary viewpoint of neoplastic existence above species level approximates the process across geological eras. Such an approach includes, among others, the figuring out of the total number of species in which neoplasia has been reported. The main purpose remains to be the acknowledgment of diversity from individual to population level and the ecological stress put on them. Some questions that arise considering the above are: How many different species have been recorded with neoplasia? At what extent does Darwinian evolution shape the phenology of cancer in studied taxa? Are there any organisms that possess cancer-protective mechanisms? Moreover, a complex task like this requires seeking for footprints and elements hidden in the past. In order to do so, it is crucial to run through the fossil record and the provided data aiming to answer fundamental questions about the origin of cancer itself in the evolutionary time. One of the most important queries that has to be answered concerns the origin of neoplasia, the circumstances under which this happened and the taxa involved.

## Neoplasia in vertebrates

In this section an overall recording of reported incidence of neoplasia in vertebrate species is attempted based on the scientific journal articles. This approach excludes domesticated animals and model organisms, which are used for all kinds of research experiments. Our focus is mainly restricted on species that live in their natural environment or protected areas.

Cancer appears to be no exception to most animal species and definitely it is not a “privilege” for some. On the contrary, incidents of neoplasia have been reported in a plethora of species from several habitats (Table 1), covering a large number of vertebrate families (Table 2). The diversity of organisms, which have been reported with neoplasia, is spread in many different habitats, each of them applying differential and particular pressure on species performance.

**Table 1:** Vertebrate taxa bearing neoplastic incidents, categorized according to their habitat

Habitat	Families (species)
Terrestrial	71 (204)
Aquatic	42 (87)
Semi-aquatic	7 (11)
Sum	120 (302)

**Table 2:** Classification of vertebrate species that have been reported with neoplasia

Class	Order	Family	Species	Common name	
Mammalia	Artiodactyla	Monodontidae	*Delphinapterus leucas*	Beluga whale	[1, 4–8]
			*Monodon monoceros*	Narwhal	[4, 6]
		Physeteridae	*Physeter macrocephalus*	Sperm whale	[4, 6]
		Phocoenidae	*Phocoena spinipinnis*	Burmeister’s porpoise	[4, 6]
			*Phocoena phocoena*	Harbor porpoise	[4, 6, 7]
			*Neophocaena phocaenoides*	Finless porpoise	[4]
			*Mirounga angustirostris*	Northern elephant seal	[4, 7]
			*Mirounga leonina*	Southern elephant seal	[4]
		Balaenopteridae	*Balaenoptera physalis*	Fin whale	[4, 7]
			*Balaenoptera borealis*	Sei whale	[4]
			*Balaenoptera musculus*	Blue whale	[4, 7]
			*Megaptera novaeangliae*	Humpback whale	[4]
		Balaenidae	*Balaena mysticetus*	Bowhead whale	[4]
		Ziphiidae	*Mesoplodon densirostris*	Blainsvilles beaked whale	[4]
		Kogiidae	*Kogia breviceps*	Pygmy sperm whale	[4]
Mammalia	Artiodactyla	Delphinidae	*Delphinus delphis*	Common dolphin	[4, 7]
			*Lagenorhynchus obscurus*	Dusky dolphin	[4, 6]
			*Lagenorhynchus acutus*	Atlantic white-sided dolphin	[4, 6]
			*Lagenorhynchus obliquidens*	Pacific white-sided dolphin	[4, 7]
			*Tursiops truncatus*	Bottlenose dolphin	[4, 6–8]
			*Stenella coeruleoalba*	Striped dolphin	[4]
			*Stenella attenuata*	Pantropical spotted dolphin	[4, 7]
			*Stenella frontalis*	Atlantic spotted dolphin	[4]
			*Globicephala melaena*	Pilot whale	[4]
			*Globicephala macrorhyncus*	Short finned pilot whale	[7]
		Iniidae	*Inia geoffrensis*	Amazon river dolphin	[4, 7]
			*Orcinus orca*	Killer whale	[4, 6, 7]
		Hippopotamidae	*Hippopotamus amphibius*	Hippopotamus	[34]
			*Choeropsis liberiensis*	Pygmy hippopotamus	[34]
		Giraffidae	*Giraffa camelopardalis*	Giraffe	[8]
Mammalia	Artiodactyla	Cervidae	*Odocoileus virginianus*	White-tailed deer	[8, 35]
			*Dama dama*	Fallow deer	[8]
			*Capreolus capreolus*	Roe deer	[7, 8]
			*Elaphurus davidianus*	Pere David’s deer	[7]
		Bovidae	*Bubalus arnee*	Wild water buffalo	[8]
			*Capra nubiana*	Nubian ibex	[7]
			*Ovis canadensis*	Bighorn sheep	[8]
		Camelidae	*Camelus dromedaries*	Arabian camel	[7]
			*Lama glama*	Llama	[36]
	Perissodactyla	Equidae	*Equus zebra*	Zebra	[8]
	Carnivora	Otariidae	*Zalophus californianus*	Sea lion	[4–8]
			*Eumetopias jubatus*	Steller sea lion	[4]
			*Callorhinus ursinus*	Fur seal	[4]
			*Arctocephalus pusillus*	Brown fur seal	[4]
			*Arctocephalus australis*	South American fur seal	[4]
Mammalia	Carnivora	Phocidae	*Halichoerus grypus*	Gray seal	[4]
			*Phoca vitulina geronimensis*	Harbor seal	[4]
			*Phoca hispida*	Ringed seal	[4]
			*Pagophilus groenlandicus*	Harp seal	[4]
		Obodenidae	*Odobenus rosmarus*	Walrus	[4]
		Ursidae	*Ursus maritimus*	Bear	[4]
			*Helarctos malayanus*	Sun bear	[37]
		Canidae	*Canis lupus*	Wolf	[6, 8]
			*Canis lupus baileyi*	Mexican gray wolf	[8, 38]
			*Canis latrans*	Coyote	[7, 8, 39]
			*Vulpes vulpes*	Red fox	[7]
			*Urocyon littoralis*	Island fox	[5–8]
		Felidae	*Panthera leo*	Lion	[7, 40–42]
			*Panthera tigris*	Tiger	[7, 40–42]
			*Panthera pardus*	Leopard	[40–42]
Mammalia	Carnivora	Felidae	*Panthera pardus japonensis*	Chinese-leopard	[41]
			*Panthera pardus nimr*	Arabian leopard	[43]
			*Panthera onca*	Jaguar	[8, 40, 42]
			*Panthera uncia*	Snow leopard	[5, 7, 41, 42]
			*Puma concolor*	Cougar	[40, 41]
			*Acinonyx jubatus*	Cheetah	[40, 44]
			*Lynx rufus*	Bobcat	[40, 42, 45]
			*Felis canadensis*	Canada lynx	[40]
		Mustelidae	*Enhydra lutris*	Sea otter	[4]
		Procyonidae	*Procyon lotor*	Raccoon	[8]
		Herpestidae	*Herpestes javanicus*	Javan mongoose	[46]
			*Suricata suricatta*	Meerkat	[7]
		Tupaiidae	*Tupaia belangeri*	Northern Treeshrew	[47]
		Viverridae	*Arctictis binturong*	Binturong (Bearcat)	[7]
	Eulipotyphla	Erinaceidae	*Atelerix albiventris*	Four-toed hedgehog	[7, 37]
Mammalia	Primates	Hominidae	*Homo sapiens sapiens*	Human	
			*Pan troglodytes*	Common chimpanzee	[11, 48]
			*Pan paniscus*	Bonobo	[11, 48]
			*Pongo pygmaeus*	Bornean orangutan	[13, 47, 48]
			*Gorilla gorilla*	Gorilla	[36, 48]
			*Gorilla beringei*	Eastern gorilla	[4]
		Cercopithecidae	*Chlorocebus aethiops sobaeus*	Green monkey	[14]
			*Cercocebus atys*	Sooty mangabey	[47]
			*Trachypithecus obscurus*	Dusky leaf monkey	[47]
			*Macaca mulatta*	Macaque	[48–50]
			*Macaca fascicularis*	Long-tailed macaque	[47, 50]
			*Macaca fuscata*	Japanese macaque	[47]
			*Macaca arctoides*	Stump-tailed macaque	[47]
			*Macaca maura*	Moor macaque	[47]
			*Macaca sinica*	Toque macaque	[47]
Mammalia	Primates	Cercopithecidae	*Papio* spp.	Baboon	[12, 47, 51]
			*Papio hamadryas*	Hamandryas baboon	[47, 48, 52]
		Atelidae	*Ateles paniscus*	Black spider monkey	[47]
		Galagidae	*Otolemur garnetti*	Northern greater galago	[25]
		Cebidae	*Saimiri sciureus*	Common squirrel monkey	[47, 50]
			*Sapajus apella*	Tufted capuchin	[47]
		Callitrichidae	*Callithrix jacchus*	Common marmoset	[47]
			*Saguinus fuscicollis*	Brown-mantled tamarin	[47, 48]
			*Saguinus oedipus*	Cotton-top tamarin	[13]
			*Saguinus fuscicollis* ssp. *leucogenys*	Andean Saddle-back Tamarin	[47]
		Lemuridae	*Lemur catta*	Ring-tailed lemur	[53]
		Aotidae	*Aotus trivirgatus*	Three-striped night monkey	[53]
		Hylobatidae	*Hylobater lar*	Lar gibbon	[13]
		Daubentoniidae	*Daubentonia madagascariensis*	Aye–Aye	[53]
	Dasyuromorphia	Dasyuridae	*Dasyuroides byrnei*	Kowari	[37]
Mammalia	Dasyuromorphia	Dasyuridae	*Dasyurus viverrinus*	Eastern quoll	[37]
			*Dasyurus maculatus*	Tiger quoll	[37]
			*Dasyurus hallucatus*	Northern quoll	[37]
			*Sarcophilus harisii*	Tasmanian devil	[6–8, 15, 17]
	Monotremata	Trachyglossidae	*Tachyglossus aculeatus*	Short-beaked echidna	[54]
	Peramelemorphia	Peramelidae	*Perameles bougainville*	W. barred bandicoot	[5, 6, 8]
	Didelphimorphia	Didelphidae	*Didelphis virginiana*	Virginia opossum	[7]
			*Monodelphis domestica*	Gray short-tailed opossum	[15, 25]
	Diprotodontia	Phascolarctidae	*Phascolarctos cinereus*	Koala	[8, 55]
	Proboscidea	Elephantidae	*Elephas maximus*	Asian elephant	[7, 25]
			*Loxodonta africana*	African elephant	[1, 7, 25]
	Perissodactyla	Rhinoceratoidae	*Diceros bicornis*	Black rhinoceros	[7, 8]
			*Ceratotherium simum*	White rhinoceros	[8]
		Tapiridae	*Tapirus* sp.	Tapir	[8]
	Sirenia	Trichechidae	*Tricherus manatus*	Manatee	[4, 6, 8]
Mammalia	Rodentia	Sciuridae	*Marmota monax*	Groundhog	[6, 7]
			*Spermophilus richardsonii*	Richardson’s ground squirrel	[37]
		Echimyidae	*Myocastor coypus*	Coypu	[7]
	Lagomorpha	Leporidae	*Sylvilagus spp*	Cottontail rabbit	[7]
			*Lepus townsendii*	White-tailed jackrabbit	[7]
	Chiroptera	Pteropodidae	*Rousettus aegyptiacus*	Egyptian fruit bat	[56, 57]
		Vespertilionidae	*Antrozous pallidus*	Pallid bat	[57]
		Phyllostomidae	*Carollia perspicillata*	Seba’s short bat	[57]
Aves	Galliformes	Phasianidae	*Tympanuchus cupido attwateri*	Attwater’s prairie chicken	[6]
			*Lophura rufa*	Vieilott’s fireback pheasant	[58]
			*Phasianus colchicus*	Common pheasant	[8]
	Psittaciformes	Psittacidae	*Amazona amazonica*	Orange-winged parrot	[18]
			*Ara ararauna*	Blue-yellow macaw	[18]
			*Ara chloropterus*	Red-green macaw	[18]
Aves	Psittaciformes	Psittacidae	*Psittacus erithacus*	Grey parrot	[18]
			*Agapornis* sp.	Lovebird	[18]
			*Aratinga solstitialis*	Sun conure	[18]
			*Aratinga nenday*	Nanday conure	[18]
			*Forpus coelestris*	Pacific parrotlet	[18]
			*Brotogeris pyrrhoptera*	Grey-cheeked parakeet	[18]
		Cacatuidae	*Nymphicus hollandicus*	Cockatiel	[18]
			*Cacatua moluccensis*	Moluccan cockatoo	[18]
		Psittaculidae	*Trichoglossus moluccanus*	Rainbow lorikeet	[18]
			*Melopsittacus undulatus*	Budgerigar	[18, 59]
			*Eclectus roratus*	Eclectus parrot	[18]
			*Psittacula krameri*	Rose-ringed parakeet	[60]
	Passeriformes	Estrildidae	*Erythrura gouldiae*	Lady gouldian finch	[18]
		Cardinalidae	*Cardinalis cardinalis*	Northern cardinal	[18]
Aves	Anseriformes	Anatidae	*Anas castanea*	Chestnut teal	[18]
			*Alopochen aegyptiaca*	Egyptian goose	[18]
	Strigiformes	Strigidae	*Ninox strenua*	Powerful owl	[18]
			*Megascops* sp.	Screech owl	[18]
			*Bubo virginianus*	Great horned owl	[18, 61]
			*Bubo scandiacus*	Snowy owl	[18]
			*Strix varia*	Barred owl	[18]
	Falconiformes	Falconidae	*Falco peregrinus*	Peregrine falcon	[18]
			*Falco sparverinus*	American kestrel	[18]
	Sphenischiformes	Sphenischidae	*Spheniscus demersus*	African penguin	[62]
			*Spheniscus humboldti*	Humboldt penguin	[18]
	Procellariiformes	Procellariidae	*Fulmarus glacialis*	Northern fulmar	[8]
	Rheiformes	Rheidae	*Rhea americana araneipes*	Greater rhea	[63]
	Phoenicopterifromes	Phoenicopteridae	*Phoenicopterus ruber*	American flamingo	[64]
Reptilia	Testudines	Cheloniidae	*Chelonia mydas*	Green sea turtle	[6, 8]
			*Dermochelys coriacea*	Leatherback sea turtle	[6]
			*Lepidochelys kempii*	Kemp’s ridley sea turtle	[6]
			*Eretmochelys imbricata*	Hawksbill sea turtle	[6]
			*Caretta caretta*	Loggerhead sea turtle	[19]
		Geoemydidae	*Cuora flavomarginata*	Chinese box turtle	[65]
			*Melanochelys trijuga*	Indian black turtle	[20]
		Testudinidae	*Testudo hermanni*	Hermann’s tortoise	[19]
			*Geochelone platynota*	Burmese star tortoise	[19]
		Emydidae	*Pseudemys concinna*	River cooter	[20]
		Trionychidae	*Apalone spinifera spinifera*	Spiny soft shell turtle	[20]
			*Apalone ferox*	Florida shoftshell turtle	[19]
		Pelomedusidae	*Pelusios subniger*	African mud turtle	[20]
		Chelidae	*Chelodina longicollis*	Common snake-necked turtle	[20]
			*Chelodina oblonga*	Northern snake-necked turtle	[20]
Reptilia	Squamata	Scincidae	*Corucia zebrata*	Solomon island skink	[20]
			*Eugongylus albofasciolatus*	Solomon island ground skink	[20]
			*Eumeces fasciatus*	Five-lined skink	[20]
			*Plestiodon laticeps*	Broad-headed skink	[20]
			*Tiliqua rugosa*	Shingleback skink	[20]
		Cordylidae	*Cordylus warreni*	Warren’s girdle lizard	[20]
		Anguidae	*Pseudopus apodus*	European legless lizard	[20]
		Shinisauridae	*Shinisaurus crocodilurus*	Crocodile lizard	[20]
		Eublepharidae	*Eublepharis macularius*	Leopard gkeko	[20]
		Helodermatidae	*Heloderma horridum*	Mexican beaded lizard	[20]
			*Heloderma suspectum*	Gila monster	[20]
		Agamidae	*Pogona vitticeps*	Bearded dragon	[21]
			*Hydrosaurus amboinensis*	East Indian water lizard	[19]
		Iguanidae	*Iguana iguana*	Green iguana	[19, 20]
			*Ctenosaura pectinata*	Mexican spiny-tailed iguana	[20]
Reptilia	Squamata	Varanidae	*Varanus exanthematicus*	Savannah monitor	[19]
			*Varanus timorensis*	Spotted tree monitor	[13]
		Viperidae	*Vipera xanthina*	Ottoman viper	[66]
			*Macrovipera lebetina*	Lebetine viper	[20]
			*Bitis gabonica*	Gaboon viper	[66]
			*Bitis nasicornis*	Rhinoceros viper	[20]
			*Bitis gabonica rhinoceros*	West African gaboon viper	[20]
			*Bothrops alternatus*	Urutu	[66]
			*Sistrurus miliarius miliarius*	Pygmy rattlesnake	[20]
			*Crotalus horridus*	Timber rattlesnake	[66]
			*Crotalus adamanteus*	Eastern diamondback rattlesnake	[20]
			*Crotalus lepidus*	Rock rattlesnake	[20]
			*Crotalus atrox*	Western diamondback rattlesnake	[66]
			*Crotalus scutulatus*	Mojave rattlesnake	[66]
			*Crotalus simus*	Yucatan neotropical rattlesnake	[66]
Reptilia	Squamata	Viperidae	*Antheris* sp.	Bush viper	[66]
			*Bothriechis lateralis*	Side-striped viper	[66]
			*Agkistrodon piscivorus*	Cottonmouth	[20, 66]
			*Agkistrodon contortrix laticinctus*	Broad-banded copperhead	[66]
			*Agkistrodon contortrix*	Copperhead	[66]
		Colubridae	*Pantherophis guttacus*	Corn snake	[20, 21, 66]
			*Elaphe (Pantherophis) obsoleta*	Black rat snake	[20, 66]
			*Pantherophis bairdi*	Baird’s ratsnake	[20]
			*Pituophis melanoleucus*	Pine snake	[20, 66]
			*Pituophis deppei*	Mexican bull snake	[20]
			*Pituophis catenifer sayi*	Bull snake	[20]
			*Coluber constrictor*	Black racer	[20, 66]
			*Lampropeltis elapsoides*	Scarlet kingsnake	[20, 66]
			*Lampropeltis calligaster*	Yellow-bellied kingsnake	[66]
Reptilia	Squamata	Colubridae	*Lampropeltis getula*	Kingsnake	[19–21]
			*Heterodon kennerlyi*	Mexican hognose snake	[66]
			*Heterodon platirhinos*	Eastern hognose snake	[20]
			*Drymarchon couperi*	Eastern indigo snake	[66]
			*Thamnophis sauritus*	Ribbon snake	[21]
			*Thamnophis radix*	Plains garter snake	[20]
			*Thamnophis sirtalis*	Common garter snake	[21]
			*Clelia clelia*	Mussurana	[20]
			*Gonyosoma oxycephalum*	Arboreal ratsnake	[20]
			*Natrix natrix*	Grass snake	[21]
		Boidae	*Boa constrictor*	Common boa	[21]
			*Corallus hortulanus*	Amazon tree boa	[66]
			*Eunectes murinus*	Common anaconda	[19]
			*Eunectes notaeus*	Yellow anaconda	[20]
Reptilia	Squamata	Boidae	*Epicrates cenchria*	Rainbow boa	[20]
			*Epicrates chrysogaster*	Turks island boa	[20]
			*Candoia bibroni*	Pacific tree boa	[20]
			*Lichanura trivirgata*	Rosy boa	[20]
			*Chilabothrus subflavus*	Jamaican boa	[21]
		Elapidae	*Pseudechis australis*	King brown snake	[66]
			*Aspidelaps scutatus*	Shield-nosed cobra	[66]
			*Dendroaspis polylepis*	Black mamba	[66]
			*Naja haje*	Egyptian cobra	[66]
			*Naja melanoleuca*	Forest cobra	[20]
			*Naja nivea*	Cape cobra	[20]
		Pythonidae	*Morelia spilota*	Carpet python	[21, 66]
			*Python regius*	Ball python	[20]
			*Bothrochilus albertisii*	D’albertis python	[20]
Reptilia	Squamata	Pythonidae	*Aspidites melanocephalus*	Black-headed python	[21]
		Loxocemidae	*Loxocemus bicolor*	Mexican python	[20]
		Lamprophiidae	*Boaedon fuliginosus*	African house snake	[21]
	Crocodilia	Crocodilidae	*Crocodylus porosus*	Saltwater crocodile	[67]
			*Crocodylus niloticus*	Nile crocodile	[67]
			*Crocodylus acutus*	American crocodile	[67]
			*Crocodylus siamensis*	Siamese crocodile	[67]
		Alligatoridae	*Alligator mississippiensis*	American alligator	[67]
Amphibia	Anura	Ranidae	*Rana pipiens*	Northern leopard frog	[6]
		Hylidae	*Trachycephalus resinifictrix*	Golden-eye tree frog	[6, 68]
	Caudata	Salamandridae	*Triturus cristatus*	N. crested newt	[32]
Actinopterygii	Salmoniformes	Salmonidae	*Salmo salar*	Salmon	[6, 8]
			*Salvelinus fontinalis*	Brook trout	[69]
			*Salvelinus namaycush*	Lake trout	[69]
Actinopterygii	Salmoniformes	Salmonidae	*Oncorhynchus tshawytscha*	Chinook salmon	[69]
			*Oncorhynchus keta*	Chum salmon	[69]
			*Oncorhynchus masou*	Masu salmon	[69]
	Pleuronectiformes	Pleuronectidae	*Parophrys vetulus*	English sole	[6]
	Anguilliformes	Anguillidae	*Anguilla japonica*	Japanese eel	[6]
	Gadiformes	Gadidae	*Melanogrammus aeglefinus*	Haddock	[69]
	Scorpaeniformes	Sebastidae	*Sebastolobus alascanus*	Shortspine thornyhead	[69]
	Perciformes	Percidae	*Sander vitreus*	Walleye	[6]
		Sciaenidae	*Micropogonias furnieri*	Whitemouth croaker	[69]
		Labridae	*Tautogolabrus adspersus*	Cunner	[69]
		Sparidae	*Sparus aurata*	Gilthead seabream	[69]
			*Acanthopagrus bifasciatus*	Twobar seabream	[69]
		Cichlidae	*Pterophyllum scalare*	Freshwater angelfish	[69]
			*Cichla monoculus*	Peacock bass	[69]
Actinopterygii	Siluriformes	Ictaluridae	*Ictalurus punctatus*	Channel catfish	[6, 69]
			*Ameiurus melas*	Black bullhead	[69]
			*Ameiurus nebulosus*	Brown bullhead	[69]
	Cypriniformes	Catostomidae	*Catostomus commersonii*	White sucker	[6]
	Cyprinodontiformes	Cyprinodontidae	*Cyprinodon variegatus*	Sheepshead minnow	[69]
			*Cyprinus caprio caprio*	Common carp	[69]
		Poeciliidae	*Xiphophorus variatus*	Variatus platy	[70]
Chondrichthyes	Lamniformes	Odontaspididae	*Carcharias taurus*	Tiger shark	[69–71]
		Lamnidae	*Carcharodon carcharias*	White shark	[10]
	Carcharhiniformes	Carcharhinidae	*Prionace glauca*	Blue shark	[69–72]
			*Carcharhinus leucas*	Bull shark	[70]
			*Carcharhinus limbatus*	Blacktip shark	[70, 71]
			*Carcharhinus brachyurus*	Cooper shark	[10]
		Sphyrnidae	*Sphyrna tiburo*	Bonnethead	[71]
Chondrichthyes	Lamniformes	Triakidae	*Mustelus canis*	Dusky smooth-hound	[70]
		Scyliorhinidae	*Scyliorhinus canicula*	Lesser spotted shark	[69, 70]
	Orectolobiformes	Hemiscylliidae	*Chiloscyllium plagiosum*	Whitespotted bamboo shark	[9]
		Ginglymostomatidae	*Ginglymostoma cirratum*	Nurse shark	[70, 71]
			*Nebrius ferrugineus*	Tawny nurse shark	[70]
		Stegostomatidae	*Stegostoma fasciatum*	Zebra shark	[7, 70]
	Squaliformes	Squalidae	*Squalus acanthias*	Spiny dogfish	[71]
	Rajifomes	Rajidae	*Raja clavata*	Thornback ray	[69, 70, 72]
			*Raja batis*	Common skate	[70]
	Myliobatiformes	Urotrygonidae	*Urobatis halleri*	Round stingray	[72]
		Potamotrygonidae	*Potamotrygon motoro*	Ocellate river stringray	[71]

### Aquatic habitats

Despite the fact that neoplastic incidents have been reported in about 40 distinct species of marine mammals, classified in more than 10 families (Table 2), the frequency in these taxa seems to be low [4]. Most of the marine mammals follow the general pattern of marine vertebrates. However, dolphins (family: Delphinidae) are those mammals in which neoplasia has been reported with greater frequency compared to other marine families [4]. Special reference has to be made in the case of beluga whale (*Delphinapterus leucas*) and its populations which are found at St. Lawrence River in Canada [1, 4–8]. In populations of this area, the incidence of cancer is approaching or even exceeding that of humans [7] while its rate is going up to 37% [4]. Exploring further the aquatic habitats, our attention moves forward to the class of Chondrichthyes, which includes a large group of the most effective ocean predators, i.e. sharks. Sharks, such as the great white (*Carcharodon carcharias*), exhibit low incidence of neoplasia [9, 10].

### Terrestrial habitats

Moving from the aquatic environment to land, animal diversity is spreading in a variety of habitats while there is no specific pattern considering the presence of neoplasia. In primates, two of the most known groups, chimpanzees and baboons, exhibit a lot of similarities about the appearance of neoplasia [11, 12]. In the past, it was thought that cancer was unusual in great apes, but, nowadays, it is becoming more and more of a conventional occurrence [see also 11]. The appearance of neoplasia is largely related to the older age classes in both hominid and non-hominid primates [see also 11, 12]. So, the older a chimpanzee is, the more likely is to develop a neoplastic event. Moreover, an interesting fact is that female individuals appear to be more vulnerable to cancer than the males [11]. This situation is similar in non-hominid primates, such as Old World monkeys [12–14].

A special case in the terrestrial habitat is the Tasmanian devil (*Sarcophilus harrisii*) [6–8, 15–17], the largest carnivorous marsupial. The Tasmanian devil is autochthonous in Tasmania and the observed tumors were identified as a particular type of contagious cancer, which is known as DFTD (Devil Facial Tumor Disease) [6–8, 15–17]. DFTD generally affects facial tissues, jaws and neck, and causes significant deformation of their soft parts [6]. The disease is transmitted through biting during social interactions and in most cases leads to death within six months from the first appearance of symptoms [17].

Across the diverse class of birds, neoplasia is reported in 34 species sorted in 13 families (Table 2). Neoplasia is more common in Psittaciformes, followed by Galliformes, Strigiformes and Falconiformes [18]. The dominant presence in the order of Psittaciformes may be the outcome of the observed longevity among the species comprising this order [18]. It is noteworthy that all families have different types of neoplasia but, as a pattern, lymphoma is more common followed by carcinoma and adenocarcinoma [18].

The reported neoplasia in reptiles (class: Reptilia) is unusual to rare compared to that for mammalian and avian classes [19]. In turtles (order: Testudines), the frequency of reported neoplasia and metastasis, in both terrestrial and aquatic species, appears to be rare [19, 20]. In lizards we have no sound evidence that the frequency of neoplastic events is considerable in wild populations. On the other hand, the percentage of reports in captive populations has increased over the recent years from 0.7% to 5.9% [20]. Snakes are mostly reported with liver and skin cancer [20, 21] but no specific pattern has been identified so far.

## How back in time does neoplasia go?—Fossil record

Cancer is present in many species throughout the animal kingdom, spreading out in several families of six vertebrate classes. Nevertheless, the following question arises. How old are these incidents of neoplasia and under which conditions did they appear? Extended evidence is related to dinosaurs, which had been dominating our planet for about 185 million years.

The fossil record reveals that neoplastic incidents have been recorded in several dinosaur species of the Mesozoic Era [22, 23]; most of the evidences refer to tumors observed in bones. At first, cancerous tumors were considered to be rare as, initially, their appearance was restricted to the Hadrosauridae [22]. Such an example was the presence of an ameloblastoma neoplasm in the lower jaw of a specimen referred as *Telmatosaurus transsylvanicus* in the early Cretaceous [23]. A more extensive and meticulous study of the fossil record has led to the identification of more neoplastic types in different taxa. The first presence of neoplasia, apart from the Hadrosaurid family, was found in a dinosaur fossil record classified in the Titanosaurs, in Brazil [22]. Furthermore, metastatic cancer and cases of osteoma have been found in the fossil record of Mosasaurs, a group of large marine reptiles, of the Jurassic period [24]. Because of the intense neoplasia presence in the Hadrosaurid group, compared with other taxa, this possible pattern may indicate a genetic propensity or, alternatively, reflect specific environmental stress affecting this target group [24].

The whole data set, the different disciplines and the large complexity of the evolutionary nature of cancer contribute to one aspect, which is answering a basic question that mostly precedes the rest: Could cancer eventually be considered as another evolutionary phenomenon that contributes to the formation or shaping-up of species?

## Molecular tolerance, durability and resistance

Assuming that every healthy cell has similar endogenous risk to bypass the control/repair mechanisms and paths, and therefore, accumulate mutations in its DNA, the animals of larger body mass/size and longevity, such as elephants and whales [4, 7], should have a higher risk of cancer than smaller ones [25]. However, the data so far tend to reach to a threshold, a phenomenon called Peto’s paradox, supporting that the incidence of cancer at the species level is not related to the number of body cells or lifespan [1, 2, 26, 27]. This trend is confirmed by the fact that there have been only few cases of cancer in whales, while at the same time, carcinogenesis has been very often reported in many other smaller mammals [7]. Therefore, a major question in cancer biology arises: How much and in what ways do animals protect themselves from such pathogeny?

A gene, known as TP53, is found in many animal genomes and in most malignant cases is either damaged or inactive [25]. Under normal conditions, the gene encodes a tumor suppressor protein [25, 28] that senses when DNA is damaged or a cell is under stress [25]. In such occasions, the produced protein either slows the cell growth (while the damage is still under repair) or triggers cell death if the stress exceeds a tolerable threshold [25]. Large animals, such as elephants, may potentially reduce cancer risk by having extra copies of TP53 [8, 25] or other genes that encode tumor suppressor proteins [25, 29]. In the African elephant (*Loxodonta africana*) genome, it has been confirmed that there is a set of a single TP53 gene and 19 retrogenes (TP53RTG), a number much higher than that of related species in the Proboscidea order, extinct or not. Elephant cells have an increased response to DNA-damage which is mediated by a hyperactive TP53 signaling pathway, depending on the number of copies of retrogenes [25].

Spontaneous neoplasia is apparently different, both by cause and pathogenicity, in different mammalian species [7]. Some mammals, however, possess very special phenotypes which are equipped with important traits for survival [28]. Major examples are two phylogenetically distant species of mole rats, the blind *Spalax* spp. and the naked *Heterocephalus glaber* [1, 25, 28, 30]. The blind mole rat (*Spalax* spp.) has been found to show an extraordinary tolerance to hypoxia, cancer-resistance and longevity despite its small body size [28, 30]. It is remarkable that *Spalax* is the only genus in which no malignant neoplasia has been detected in thousands of individuals examined in the past 40 years of research [28]. Moreover, with only very few exceptions, *Spalax* spp. displays a remarkable tolerance to chemically induced carcinogenesis in vivo, while its fibroblasts inhibit cancer growth in vitro [28, 30]. The naked mole rat *H. glaber*, similar to *Spalax* spp., shows adaptations to hypoxic stress and an in vitro ability to inhibit cancer cell growth [30].

The interpretation of these special phenotypic traits is hidden in certain regions of the genome. Genome sequencing of *Spalax* spp. and genome-transcriptome analysis have revealed interesting genomic features, which are potentially the basis of some of the observed adaptive traits [30]. Some of them include high rates of DNA editing (repair mechanism), reduced chromosomal rearrangements, adaptation to hypoxic/hypercapnic conditions by positive selection of respiratory proteins and reduced sensitivity to hypercapnia-induced acid pain [30].

Contrary to mammals, in which spontaneous neoplasia is present in many families, in wild amphibian populations it is extremely rare [31, 32] (Table 2). Amphibians, also, appear to be resistant to chemically induced malignant neoplasms [31]. A particular difference between amphibians and mammals may be the requirement of mammalian cells to enter the cell cycle before programmed cell death by apoptosis. On the other hand, amphibian cells have the ability to undergo apoptosis immediately in response to antigens that induce or, even, promote carcinogenesis. This feature could serve as a cytoprotective mechanism reducing the sensitivity or availability of cells that could be stimulated and become malignant [31]. More protective mechanisms and traits may be hidden in this group of taxa, like an important antimicrobial peptide family called dermaseptin that needs additional research. Dermaseptin-PH, which is a specific peptide derived from the skin secretion of a frog (*Pithecopus hypochondrialis*) specimen, exhibits a wide range of antimicrobial and anticancer activities [33]. However, any antimicrobial peptide is not necessarily an anticancer agent.

## Conclusions

The necessity of understanding evolutionary mechanisms as driving forces matched with cancer as a potential result of natural selection, have triggered many scientists to search beyond humans clarifying the importance of vertebrate populations in this quest. A variety of vertebrate species have been reported over the years with neoplasia and despite the fact that the total number of recorded incidents seems to be low (Table 1), it is very important to focus on the following fact: the crushing majority of studied cases revealed some form of cancer, which became lethal while cases in which neoplasia has not been identified are only exceptions. Looking into the evolutionary perspective of cancer, much more attention should be focused on the past. The Mesozoic Era offers a plethora of neoplastic incidents, which may have a catalytic contribution to this endeavor. The rich fossil record contains important evidence for possible evolutionary paths and traits and many more are expected in the future if such a study is prioritized. Therefore, data from the fossil record should be utilized to unravel the conditions and/or pressures of the geo-chronological period, which may attribute to cancer its potential impacts on the formation and shaping-up of species.

The collected data tend to define cancer as a common phenomenon through the animal kingdom. However, there are cases in which specific taxa possess features capable of defending this pathogeny. Both mole rats cited above, *Spalax* spp. and *Heterocephalus glaber* [1, 25, 28, 30], show some remarkable tolerance and resistance to cancer due to specific genes in their genomes. Moreover, the low incidence of cancer in amphibians [31, 32] and the presence of some peptides with interesting properties [33] point out new research insights. These cases, among others, indicate the prospects for investigating the tumor suppressive mechanisms and cancer-resistant phenology in phylogenetically distal taxa. Their potential relationship with conserved elements in human genome and their significance in biomedical procedures should be addressed as holistically as possible.

Despite the growing interest of the scientific community towards the ecological and evolutionary background of neoplasia in wild populations, the list of relevant literature remains limited [6, 8]. To acquire safer and clearer ascertainments, it is essential to intensify these efforts since the benefits of studying wild population dynamics in their own habitats are multiple. This is expected to provide crucial evidence and answers to the very primary queries on the evolutionary nature of cancer while its effects on individual and population level disclose the onset of neoplastic phenomena through time, as major factors in the survival and shaping up of species.

## Data Availability

Not applicable.

## References

[1] Caulin AF, Maley CC (2011). Peto’s Paradox: evolution’s prescription for cancer prevention. Trends Ecol Evol.

[2] Nunney L, Maley CC, Breen M, Hochberg ME, Schiffman JD (2015). Peto’s paradox and the promise of comparative oncology. Trans R Soc Lond B Biol Sci..

[3] Merlo LM, Pepper JW, Reid BJ, Maley CC (2006). Cancer as an evolutionary and ecological process. Nat Rev Cancer.

[4] Newman SJ, Smith SA (2006). Marine mammal neoplasia: a review. Vet Pathol.

[5] Giraudeau M, Sepp T, Ujvari B, Ewald PW, Thomas F (2018). Human activities might influence oncogenic processes in wild animal populations. Nat Ecol Evol..

[6] McAloose D, Newton AL (2009). Wildlife cancer: a conservation perspective. Nat Rev Cancer.

[7] Nagy JD, Victor EM, Cropper JH (2007). Why don’t all whales have cancer? A novel hypothesis resolving Peto’s paradox. Integr Comp Bio..

[8] Pesavento PA, Agnew D, Keel MK, Woolard KD (2018). Cancer in wildlife: patterns of emergence. Nat Rev Cancer.

[9] Culp BE, Haulena M, Britt K, Evans H, Raverty S (2017). Squamous cell carcinoma of the rostral maxilla in an adult captive whitespotted bamboo shark (*Chiloscyllium plagiosum*). J Zoo Wildl Med..

[10] Huveneers C, Klebe S, Fox A, Bruce B, Robbins R, Borucinska JD (2016). First histological examination of a neoplastic lesion from a free-swimming white shark, *Carcharodon carcharias* L.. J Fish Dis..

[11] Brown SI, Anderson DC, Dick EJ, Guardado-Mendoza R, Garcia AP, Hubbard GD (2009). Neoplasia in the chimpanzee (*Pan* spp.). J Med Primatol.

[12] Cianciolo RE, Butler SD, Eggers JS, Dick EJ, Leland MM, de la Garza M (2007). Spontaneous neoplasia in the baboon (*Papio* spp.). J Med Primatol.

[13] Ikpatt OF, Reavill D, Chatfield J, Clubb S, Rosenblatt JD, Fonte G (2014). Diagnosis and treatment of diffuse large b-cell lymphoma in an orangutan (*Pongo pygmaeus*). J Zoo Wildl Med..

[14] Valentine MJ, Beierschmitt A, Delay J, Callanan JJ (2016). Uterine angioleiomyoma in an African green monkey (*Chlorocebus aethiops sabaeus*). J Med Primatol.

[15] Gallus S, Hallström BM, Kumar V, Dodt WG, Janke A, Schumann GG (2015). Evolutionary histories of transposable elements in the genome of the largest living marsupial carnivore, the Tasmanian devil. Mol Biol Evol.

[16] Kokko H, Hochberg ME (2015). Towards cancer-aware life-history modelling. Philos Trans R Soc Lond B Biol Sci.

[17] Ujvari B, Papenfuss AT, Belov K (2016). Transmissible cancers in an evolutionary context. BioEssays.

[18] Nemeth NM, Gonzalez-Astudillo V, Oesterle PT, Howerth EW (2016). A 5-year retrospective review of avian diseases diagnosed at the Department of Pathology, University of Georgia. J Comp Pathol..

[19] Orós J, Torrent A, Monteros AEDL, Calabuig P, Déniz S, Tucker S (2001). Multicentric lymphoblastic lymphoma in a loggerhead sea turtle (*Caretta caretta*). Vet Pathol.

[20] Sykes JM, Trupkiewicz JG (2006). Reptile neoplasia at the philadelphia zoological garden, 1901–2002. J Zoo Wildl Med..

[21] Dietz J, Heckers KO, Aupperle H, Pees M (2016). Cutaneous and subcutaneous soft tissue tumours in snakes: a retrospective study of 33 cases. J Comp Pathol.

[22] Barbosa FHDS, Da Costa Pereira PVLG, Bergqvist PL, Rothschild BM (2016). Multiple neoplasms in a single sauropod dinosaur from the Upper Cretaceous of Brazil. Cretac Res.

[23] Dumbravă MD, Rothschild BM, Weishampel DB, Csiki-Sava Z, Andrei RA, Acheson KA (2016). A dinosaurian facial deformity and the first occurrence of ameloblastoma in the fossil record. Sci Rep..

[24] Rehemtulla A (2010). Dinosaurs and ancient civilizations: reflections on the treatment of cancer. Neoplasia..

[25] Sulak M, Fong L, Mika K, Chigurupati S, Yon L, Mongan NP (2016). TP53 copy number expansion is associated with the evolution of increased body size and an enhanced DNA damage response in elephants. eLife..

[26] Roche B, Hochberg ME, Caulin AF, Maley CC, Gatenby RA, Misse D (2012). Natural resistance to cancers: a Darwinian hypothesis to explain Peto’s paradox. BMC Cancer..

[27] Roche B, Sprouffske K, Hbid H, Missé D, Thomas F (2012). Petos paradox revisited: theoretical evolutionary dynamics of cancer in wild populations. Evol Appl.

[28] Schmidt H, Malik A, Bicker A, Poetzsch G, Avivi A, Shams I (2017). Hypoxia tolerance, longevity and cancer-resistance in the mole rat Spalax—a liver transcriptomics approach. Sci Rep..

[29] Casás-Selves M, DeGregori J (2011). How cancer shapes evolution and how evolution shapes cancer. Evolution.

[30] Domankevich V, Opatowsky Y, Malik A, Korol AB, Frenkel Z, Manov I (2016). Adaptive patterns in the p53 protein sequence of the hypoxia- and cancer-tolerant blind mole rat *Spalax*. BMC Evol Biol.

[31] Taylor SJ, Johnson RO, Ruben LN, Clothier RH (2003). Splenic lymphocytes of adult Xenopus respond differentially to PMA in vitro by either dying or dividing: significance for cancer resistance in this species. Apoptosis.

[32] Zilakos NP, Tsonis PA (1991). A spontaneous melanoma-like tumor in the adult newt *Triturus cristatus*. Tumor Biol..

[33] Huang L, Chen D, Wang L, Lin C, Ma C, Xi X (2017). Dermaseptin-PH: a novel peptide with antimicrobial and anticancer activities from the skin secretion of the South American orange-legged leaf frog, *Pithecopus* (*Phyllomedusa*) *hypochondrialis*. Molecules.

[34] Schiaffino F, Sander SJ, Bacares MEP, Barnes KJ, Kiupel M, Walsh T (2016). Cerebellar And Mesencephalon Neoplasia In A Nile Hipoppotamus (*Hippopotamus amphibious*). J Zoo Wildl Med..

[35] Kalb MM, Miller DL, Keel MK, Bowman JL (2018). Rare osteochondroma associated with natural mortality of a wild white-tailed deer (*Odocoileus virginianus*). J Wildl Dis.

[36] Olias P, Schulz E, Ehlers B, Ochs A, Mundhenk L, Klopfleisch R (2012). Metastatic endocervical adenocarcinoma in a western lowland gorilla (*Gorilla g. gorilla*)—no evidence of virus-induced carcinogenesis. J Med Primatol.

[37] Saunders R, Killick R, Barrows M, Stidworthy M (2017). Oral squamous cell carcinoma in three related kowari (*Dasyuroides byrnei*). J Comp Pathol.

[38] Federico RA, Hector FTM, Martin EPS, Jose HMG, Hector MHC (2010). Case report of malignant mammary neoplasia in Mexican gray wolf (*Canis lupus baileyi*). J Anim Vet Adv..

[39] Bernstein KS, Schelling SH (1999). Oral squamous cell carcinoma in a coyote (*Canis latrans*). J Zoo Wildl Med..

[40] Owston MA, Ramsay EC, Rotstein DS (2008). Neoplasia in felids at the Knoxville zoological gardens, 1979–2003. J Zoo Wildl Med..

[41] Pope JP, Steeil J, Ramsay EC, Reel D, Newman SJ (2016). Spontaneous proliferative and neoplastic lesions in thyroid and parathyroid glands of nondomestic felids. J Vet Diagn Invest.

[42] Sadler RA, Craig LE, Ramsay EC, Helmick K, Collins D, Garner MM (2016). Clinicopathologic features of mammary masses in captive lions (*Panthera Leo*). J Zoo Wildl Med..

[43] Baqir S, Al Azri HA, Al Rasbi KA, Mastromonaco GF, Gartley C (2014). Skin lipoma in an Arabian leopard (*Panthera paradus nimr*). Sci Vet..

[44] Lindemann DM, Carpenter JW, Almes KM, Schumacher L, Ryseff JK, Hallman M (2015). Solitary T-Cell hepatic lymphoma with large granular lymphocyte morphology in a captive cheetah (*Acinonyx Jubatus*). J Zoo Wildl Med..

[45] Sladakovic I, Burnum A, Blas-Machado U, Kelly LS, Garner BC, Holmes SP (2016). Mandibular squamous cell carcinoma in a bobcat (*Lynx rufus*). J Zoo Wildl Med..

[46] Dawood K (2012). Mammary gland adenocarcinoma in three small Indian mongooses (*Herpestes javanicus*). J Anim Vet Adv..

[47] Haddad JL, Dick EJ, Guardado-Mendoza R, Hubbard GB (2009). Spontaneous squamous cell carcinomas in 13 baboons, a first report in a spider monkey, and a review of the non-human primate literature. J Med Primatol.

[48] Stringer EM, Voe RSD, Valea F, Toma S, Mulvaney G, Pruitt A (2010). Medical and surgical management of reproductive neoplasia in two western lowland gorillas (*Gorilla gorilla gorilla*). J Med Primatol.

[49] Cazzini P, Krimer PM, Williams-Fritze MJ, Butler AM, Blas-Machado U (2014). Spontaneous chronic T-cell leukemia in a male rhesus macaque (*Macaca mulatta*). J Vet Diagn Invest.

[50] Roberts BM, Chumpolkulwong K, Tayamun S, Inamnuay L, Rungsipipat A, Lombardini ED (2014). Mammary carcinoma in a male rhesus macaque (*Macaca mulatta*): histopathology and immunohistochemistry of ductal carcinomain situ. J Med Primatol.

[51] Owston MA, Larue MK, Dick EJ, Ambrus A, Porter BF (2016). Pancreatic neuroendocrine tumors in twelve baboons (*Papio* spp.). J Med Primatol.

[52] Mezzles MJ, Dick EJ, Owston MA, Bauer C (2015). Osteosarcoma in baboons (*Papio* spp.). Comp Med.

[53] Barbon AR, Cowen R, Knott C, Hughes K, Allinson K, Williams CV (2018). Neoplasia in three aye-ayes (*Daubentonia madagascariensis*). J Comp Pathol.

[54] Robey RI, Sangster C, Gabor M, Lindsay SA (2018). Soft tissue sarcoma in a short-beaked echidna (*Tachyglossus aculeatus*). Aust Vet J.

[55] Canfield PJ, Hemsley S (1996). Thymic lymphosarcoma of T cell lineage in a koala (*Phascolarctos cinereus*). Aust Vet J.

[56] Cushing AC, Ossiboff R, Buckles E, Abou-Madi N (2013). Metastatic pancreatic carcinoma and bronchioalveolar adenomas in an egyptian fruit bat (*Rousettus aegyptiacus*). J Zoo Wildl Med..

[57] Olds JE, Burrough ER, Fales-Williams AJ, Lehmkuhl A, Madson D, Patterson AJ (2015). Retrospective evaluation of cases of neoplasia in a captive population Of Egyptian fruit bats (*Rousettus aegyptiacus*). J Zoo Wildl Med..

[58] Zordan MA, Garner MM, Smedley R, Neelis D, Sánchez CR (2017). Leiomyosarcoma of the wing in a Vieilotts fireback pheasant (*Lophura rufa*). J Avian Med Surg..

[59] Youl JM, Gartrell BD (2006). Multidrug-resistant bacterial ingluvitis associated with squamous cell carcinoma in a Budgerigar (*Melopsittacus undulatus*). Vet Clin N Am Exot Anim Pract..

[60] Suedmeyer W, Henry C, Mccaw D, Boucher M (2007). Attempted photodynamic therapy against patagial squamous cell carcinoma in an african rose-ringed parakeet (*Psittacula Krameri*). J Zoo Wildl Med..

[61] Wiley JL, Whittington JK, Wilmes CM, Messick JB (2009). Chronic myelogenous leukemia in a great horned owl (*Bubo virginianus*). J Avian Med Surg..

[62] Ferrell ST, Marlar AB, Garner M, Lung NP (2006). Intralesional cisplatin chemotherapy and topical cryotherapy for the control of choanal squamous cell carcinoma in an African Penguin (*Spheniscus demersus*). J Zoo Wildl Med..

[63] Rocha PRD, Lopes LL, Arruda LPD, Pescador CA, Cruz RASD, Colodel EM (2015). Cholangiocarcinoma in an American Rhea (*Rhea americana araneipes*). Cienc Rural..

[64] Abu J, Wünschmann A, Redig PT, Feeney D (2009). Management of a cutaneous squamous cell carcinoma in an American Flamingo (*Phoenicopterus ruber*). J Avian Med Surg..

[65] Bezjian M, Diep AN, de Matos R, Schaefer D (2013). Chinese Box turtle (*Cuora flavomarginata*) with lymphoid leukemia characterized by immunohistochemical and cytochemical phenotyping. Vet Clin Pathol..

[66] Page-Karjian A, Hahne M, Leach K, Murphy H, Lock B, Rivera S (2017). Neoplasia in snakes at zoo Atlanta during 1992–2012. J Zoo Wildl Med..

[67] Hill AG, Dennis MM, Pyne M (2016). Squamous cell carcinoma with hepatic metastasis in a saltwater crocodile (*Crocodylus porosus*). Aust Vet J.

[68] López J, Barbón AR, Smithyman J, Goetz M, Marschang RE, Dastjerdi A (2016). High Prevalence of intestinal adenocarcinoma in a captive population of Amazon milk frog (*Trachycephalus resinifictrix*). J Zoo Wildl Med..

[69] Borucinska JD, Harshbarger JC, Reimschuessel R, Bogicevic T (2004). Gingival neoplasms in a captive sand tiger shark, *Carcharias taurus* (Rafinesque), and a wild-caught blue shark, *Prionace glauca* (L.). J Fish Dis.

[70] Waldoch JA, Burke SS, Ramer JC, Garner MM (2010). Melanoma in the skin of a nurse shark (*Ginglymostoma cirratum*). J Zoo Wildl Med..

[71] Jafarey YS, Berlinski RA, Hanley CS, Garner MM, Kiupel M (2015). presumptive dysgerminoma in an orange-spot freshwater stingray (*Potamotrygon motoro*). J Zoo Wildl Med..

[72] Nau MR, Gardiner DW, Nilson E, Schmitt TI, Nollens HH, Leger JS (2016). Cutaneous malignant melanoma in a Haller’s round ray *Urobatis halleri*. Dis Aquat Organ..

